# Employment Psychology of Young Migrant Workers During Coronavirus Disease 2019: A Comparative Study Between Construction Workers and Food Delivery Knights

**DOI:** 10.3389/fsoc.2022.874681

**Published:** 2022-06-28

**Authors:** Cong Xue, Chuan Zhou, Xing Su, Zhongfu Qin

**Affiliations:** ^1^Zhejiang University, Hangzhou, China; ^2^School of Microelectronics, Tianjin University, Tianjin, China; ^3^College of Civil Engineering and Architecture, Zhejiang University, Hangzhou, China

**Keywords:** migrant workers, job search, construction industry, coronavirus disease 2019, food deliver industry, employment psychology

## Abstract

The employment psychology of young migrant workers in China has changed drastically in past decades. In particular, the construction industry has been facing labor shortages and aging workforces for years, and the eruption of coronavirus disease 2019 has exacerbated the problem. In contrast, the food delivery business has grown rapidly during the pandemic with a surge in the number of food delivery knights. It is vitally important to understand the employment psychology of the young migrant workers, the main component of the workforce for both industries. The presented study conducted a comparative analysis between construction workers and food delivery knights using data from face-to-face interviews, online social media, and World Value Survey. Results showed that the two groups of young migrant workers have different employment psychology during their job selection, construction workers cared more about income, and food delivery knights paid more attention to autonomy, working environment, and family.

## Introduction

Migrant workers in China refer to those who enter the urban areas from rural areas and engage in non-agricultural labor work for 6 months or more and take non-agricultural income as the main income workers (Human Relations, 2012). Migrant workers are an important part of China's labor market. They form the main body of the labor force in traditional industries such as the construction and manufacturing industries (Wang et al., 2015). For a long time, the construction industry was the popular choice for young migrant workers because of its relatively high pay. However, there have emerged new trends in young migrant workers' job choices in recent years.

**Figure 1:**
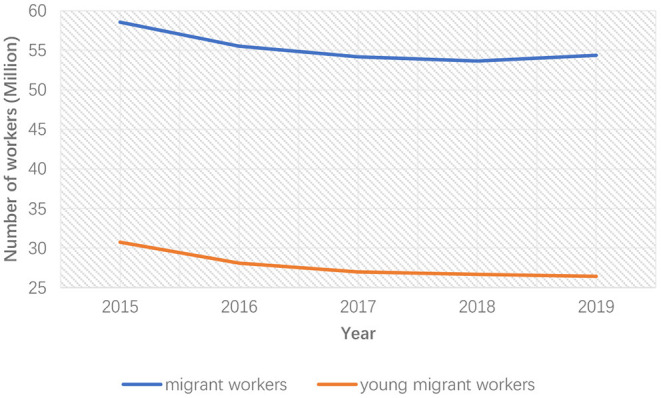
The number of migrant workers and young migrant workers in the construction industry.

**Figure 2:**
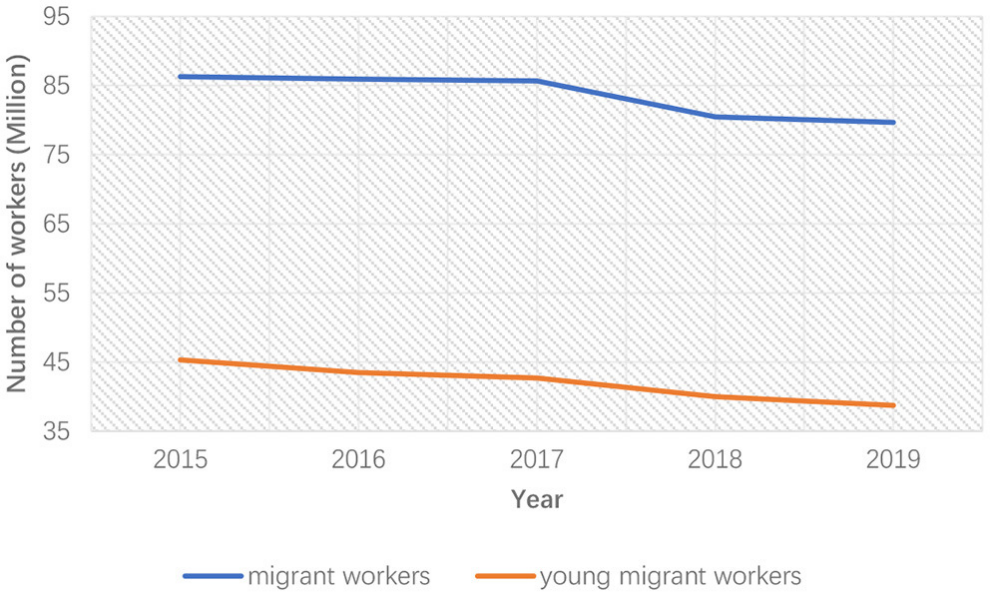
The number of migrant workers and young migrant workers in the manufacturing industry.

With the decline of China's population growth rate and the increasing population aging (Jones et al., 2010), traditional industries are struggling with labor aging and shortage in China and many other countries (Saleh, 2008). The average age of migrant workers in China comes to 40.8 years in 2019 from 33.5 in 2010. According to the data for 2019, migrant workers over 40 years old accounts for 49.4% in total, while this number in 2010 was only 12.9%. The number of migrant workers over 40 years old increased from 31 million in 2010 to 65 million in 2018 (Central Government, 2022). Figures 1, 2 show the change in the number of migrant workers and young migrant workers in the construction and manufacturing industries in recent years. The number of migrant workers in both industries experienced a large decrease in the past decade, and this trend became more obvious after 2015. Especially the number of young migrant workers, which refers to migrant workers aging from 20 to 40, decreased by 6–7 million in the construction industry and 10 million in the manufacturing industry.

**Figure 3:**
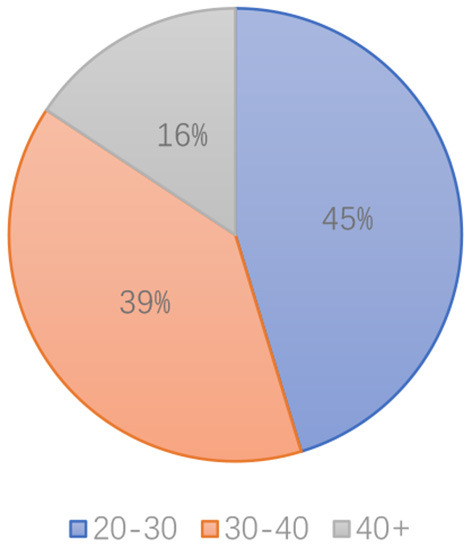
Age of riders.

In contrast, the food delivery business developed rapidly and attracted many migrant workers. In 2015, the food delivery business entered the fast lane of development. At that time, there were three major companies, Meituan, Eleme, and Baidu takeout. Meituan started its food delivery business in 2013. Eleme was founded in 2008 and acquired Baidu takeaway in 2017. Since 2017, the food delivery business has entered the monopoly stage of Eleme and Meituan. Both the market size and the number of riders have surged. In 2020, the eruption of COVID-19 promoted a significant change in the economic structure of China and the development of the food delivery business. The number of food delivery knights experienced a rise of 5.8 million in 2 months. According to the data of Meituan, many people from the construction industry turn to taking the job of riders because of COVID-19 (Meituan, 2022b). Most new riders are from 20 to 40 years old (Figure 3). Labor in the secondary industry is the biggest source of new riders. The manufacturing industry accounts for 18.6%, while the construction industry accounts for 9%.

With a growing number of young migrant workers choosing to become food delivery knights while the number in the construction industry keeps declining, it remains to be revealed what differences in employment psychology the two groups of young migrant workers have during job selection. The food delivery business demonstrates strong characteristics in both positive and negative ways. Food delivery knights' income is usually less than construction workers and much more unstable. Most of the food delivery knights do not have the “five insurances and housing provident fund” and are suffering the risk of traffic accidents. Moreover, the takeaway rider is not seen as a proper job by many people in China. The riders may have pressure from parents and relatives regarding their personal development.

On the other hand, the food delivery job has its advantages. The flexible working hour gives riders the chance to take part-time jobs. More pay for more work is also allowed in the job delivery industry. The working and living environment for food delivery knights is better because they can work in urban areas or close to home. It is critical to study the young migrant workers' employment psychology leading to their choices.

## Theoretical Framework

Job choice theory was originally outlined by Behling et al. (1968) and later articulated in the educational setting by Young et al. (1993). In classic job choice theory, many existing studies have analyzed the determinants of job choice. Oshodi et al. (2019) examined the relevant literature in the Scopus database and wrote a concise report on the factors influencing young people's career decision-making. Athanasou (2003) set out a Perceptual-Judge-mental-Reinforcement approach to job choice and induced the factors influencing job choice. The often-mentioned factors are elaborated as follows.

### Income

Koene et al. (2004) showed that economic factors could strengthen and support each other in a specific workplace, synergizing workers' emotions and behavior. Young et al. (1993) said that candidates are “economic beings” who “seek to maximize their economic status by joining the organization that is perceived as being the most economically competitive”. Mcgraw's research also suggested that “good salary” is an important factor in job choice for both men and women employees (McGraw et al., 2012).

### Working Environment

Young et al. (1989) suggested that job applicants' organizational choices are influenced by job and organizational attributes. McGraw's research showed that the workplace is the second most important job attribute (McGraw et al., 2012). An NASSP-sponsored study conducted by the Educational Research Service (ERS) reported that “job too stressful” and “too much time required” are strong barriers that discourage graduates from employment (Service, 1998).

### Personal Development

The judge stated that personal development is part of working values linked to job choice decisions (Judge and Bretz, 1992). Christensen and Wright (2011) analyzed the Effects of Public Service Motivation on Job Choice Decisions and emphasized the meaning of person-job fit. Venkataraman (2015) also stated that the care for personal development could influence people's job choices.

### Family

Miller and Spriggs (2015) reported that many employees try to obtain a work-family balance in job seeking. Basil and Basil (2008) research showed that some employees tend to “find a position close to my family”. Pas et al. (2008) reported that a family-friendly work environment is attractive, especially for women in job choice. The concern of family is an important factor in people's job selection. Nevertheless, the old generation of migrant workers in China did not attach enough importance to family (Liu et al., 2013; Gao et al., 2018; Siu and Unger, 2020).

### Psychological Needs

Wang and Liu (2019) studied the impact of psychological contracts on job mobility in the construction industry, especially for migrant construction workers in China. Zhao stated that psychological contracts reflect “individual beliefs, shaped by the organization regarding terms of an exchange agreement between individuals and their organization”. A psychological contract breach captures “the employee's perception regarding the extent to which the organization has failed to fulfill its promises or obligations” (Zhao et al., 2007). Koene and Paauwe mentioned that psychological contracts (PC) could strengthen and support each other in a specific workplace (Sonnenberg et al., 2011). Gellerman (1984) noted that the candidate would tend to choose an organization that she or he believes will most likely meet her or his psychological needs. Barlow (1965) reported that engineers' psychological needs and expectations for the jobs it has would forge ahead.

### Personal Characteristics

Larkin et al. (2007) mentioned significant differences among age groups in reasons for job choice among students. Athanasou (2003) suggested that people in different age groups tend to make different job choices. Park and Word (2012) noted that age could impact both job choice and motivation and the level of intrinsic motivation; Granovetter (2018) mentioned factors about family conditions that could not be ignored in people's job choices. Bowles and Gintis (2002) proposed that labor's status has been decided by their family background before they get onto labor markets. Kerckhoff et al. (1985) mentioned that a son's job choices greatly relate to their father. Carmichael (2000) research also demonstrated that sons' job choice is influenced by their father, and DeJong et al. (1971) reported that girls' have the same situation. Yoo and Oh (2017) research showed that family economic conditions could affect people's job choices in South Korea; Kenneth mentioned that the region of origin is an important determinant in the job choice of rural labor migrants in Shanghai (Roberts, 2018). Sidhu (2011) mentioned that culture could also affect people's choices. Kulkarni and Nithyanand (2013) reported that social influence makes sense in job choice. As to migrant workers' job choices in China, Antia reported that the new generation of migrant workers tend to attach more importance to individual development while the old generation tends to focus on the salary (Chan and Siu, 2012). Other research showed that the new generation of migrant workers has more expectations of income and psychological needs (Wong et al., 2008). Wang et al. (2013) reported that work environment, labor intensity, and social security can influence migrant workers' job choices.

During the past decade, many scholars have studied the employment psychology of young migrant workers in different regions. The job determinants for young migrant workers show some differences. However, income, working environment, personal development, psychological needs, and family are the main determinants in general. To comprehensively show the tendency of young migrant workers' job determinants, we list the results of some representative studies.

According to Table 1, young migrant workers showed some new preferences in job selection. They tend to attach less importance to income compared to the old generation. Personal development and psychological needs received more emphasis from the new generation. On the other hand, young migrant workers' concern for working conditions and family was less significant. Factors like working time flexibility and salary settlement cycle (daily, weekly, or monthly pay) were rarely mentioned. Most of the studies were conducted before 2017. In the past several years, the internet economy has grown rapidly, and the food delivery business is to give a representative example. Many post-90s and post-00s came to cities as migrant workers. Together with the influence of COVID-19 on people's lives and careers, we have a good reason to assume some changes in the young generation of migrant workers' employment psychology to be investigated.

**Table 1:** Existing research of Chinese migrant workers.

**References**	**Region**	**Questionnaire capacity**	**Age of migrant workers**	**Top 3 important determinants**
Li, 2017	Hunan, Anhui, Guizhou	751	<40	Income Personal development Family
Peng and He, 2016	Xiamen, Chongqing, Beijing, Changsha, Chengdu	248		Family Income Working environment
Ao, 2016	Henan	167	16–35	Family Personal development Income
Song, 2015	Hebei	293	19–34	Income Personal development Psychological needs
Cao, 2014	Chongqing	267	16–33	Working environment Personal development Income
Lu and Shi, 2013	Jiangsu	586	16–32	Income Working environment
Ren (2012)	Chongqing	502	16–35	Income Personal development Psychological needs
Liu, 2011	Shenzhen	298	16–30	Income Psychological needs Personal development
Luo, 2011	Guizhou	240	16–30	Family Income Personal development
Peng and Zhu, 2014	Sichuan etc.	549	16–30	Income Personal development Working environment
Ning, 2010	Changsha	150	20–34	Income Personal development Psychological needs
Li, 2010	Hunan	240	16–25	Psychological needs Income Personal development

## Hypotheses

Based on the existing studies and the difference in job characteristics between the construction industry and the food delivery business, we promote the following hypotheses.

### Hypothesis 1: The Amount and Stability of Salary Are Less Important in Job Selection for Food Delivery Knights

**Figure 4:**
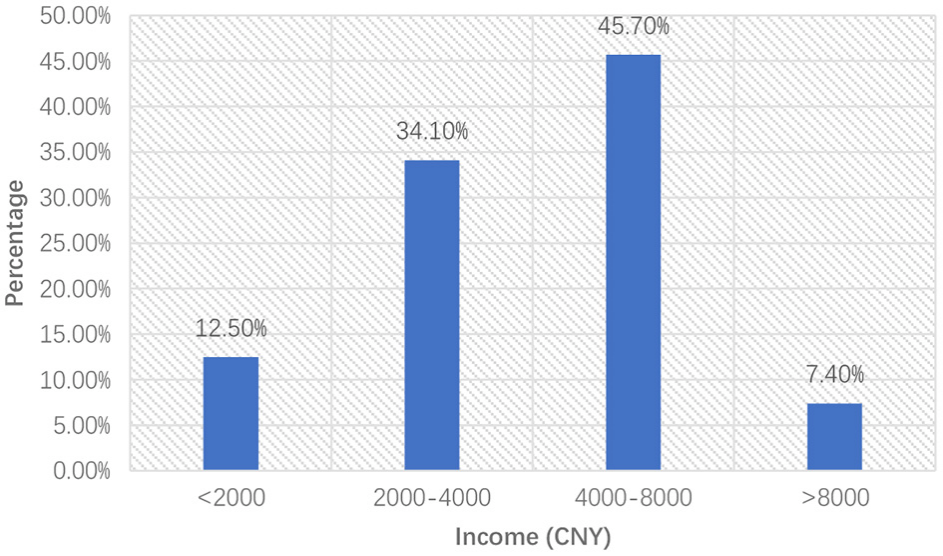
Monthly income of Meituan food delivery knights.

In traditional job choice theory, income is an important factor in people's job selection (Young et al., 1993; Koene et al., 2004; McGraw et al., 2012). Many studies also showed that income had been one of the most important job determinants for young migrant workers. Usually, jobs with good remuneration are more attractive, especially for migrant workers. However, compared to the construction industries, the food delivery business does not have a significant advantage in terms of salary. Meituan's data shows that 45.7% of food delivery knights' income ranges from ¥ 4,000 to ¥ 8,000, only 7.4% of which are more than 8,000 (Figure 4) (Meituan, 2022a).

**Figure 5:**
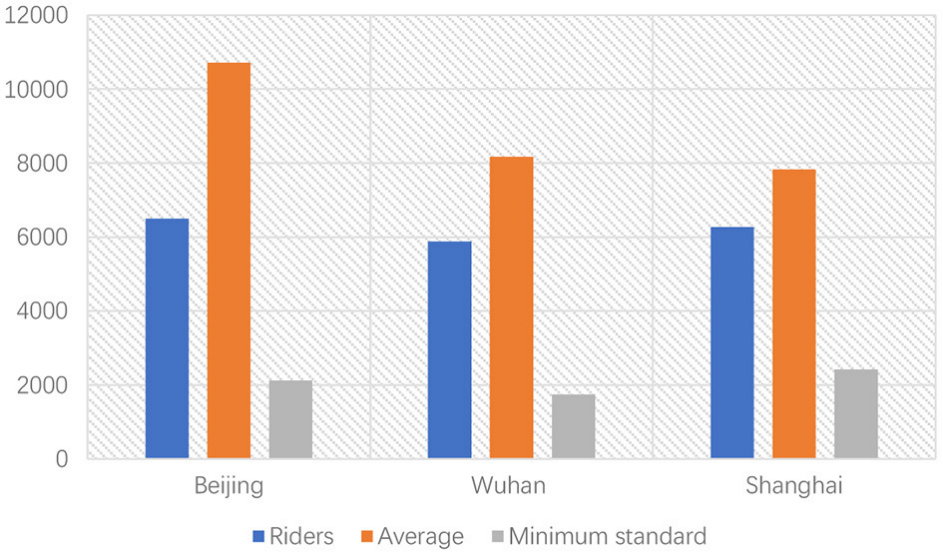
Monthly income of riders in big cities.

Meituan's data only reflect the income of full-time riders, and many researchers have doubted the accuracy. Shen surveyed food delivery knights and showed that they were in a dilemma of unstable income because of the fluctuation of orders (Jinyang, 2022). The research showed the current situation of food delivery knights in the era of “Internet Plus”, many food delivery knights' wages fluctuated in a large range and were lower than the average national wage (He and Du, 2019). Hao's research showed that in many Chinese cities, food delivery knights' average income is lower than the overall wage standard (e.g. Figure 5) (Hao, 2020). Moreover, food delivery knights do not have the compensation for overtime pay and Nightshift allowance; they do not have social insurance and cannot get compensation for injury on the job (Fuxiang et al., 2019). Considering these factors, the income of food delivery knights may be lower. As to the income of migrant workers in construction industries, data shows that the average annual wage of construction workers is more than ¥ 65,000 (Central Government, 2022).

**Figure 6:**
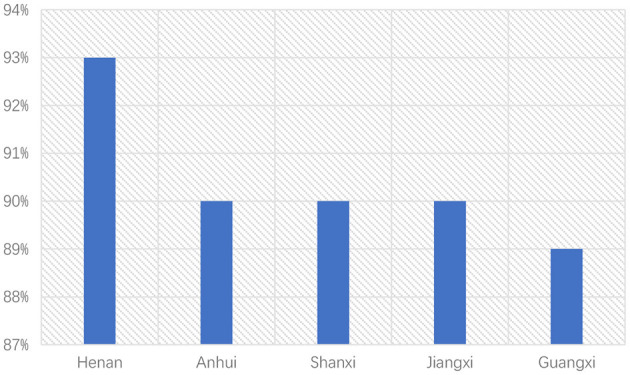
The top5 province with the most local riders.

With no obvious advantage in salary, the food delivery business may be attractive in other aspects for job-seekers. Regarding the working environment, food delivery knights can work and live in urban areas, while workers in construction industries usually can only work and live on construction sites. Besides, food delivery knights may be a good choice for people who prefer to choose a workplace close to their homes. Data shows that 53% of food delivery knights work in their hometown province. In some undeveloped provinces in China, this proportion is more than 90% (e.g. Figure 6). In big cities like Beijing and Shanghai, although the proportion of local riders is small, most riders are from neighboring regions (Meituan, 2022a).

In summary, these characteristics may imply that young migrant workers care about not only money but also life qualities and family, which shows a shift in money view. Therefore, we promote the hypothesis that young migrant workers have a different attitude to money during their job selection.

### Hypothesis 2: Job Autonomy Is More Important in Job Selection for Food Delivery Knights

**Figure 7:**
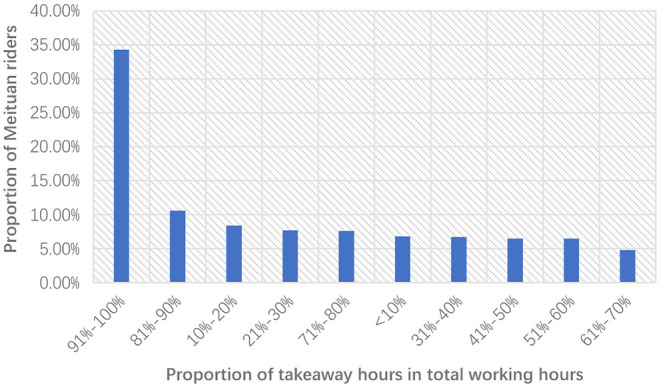
The proportion of working hours on food delivery of Meituan riders.

A specific characteristic of the food delivery business is its flexible working system. To food delivery knights, both income and working hours are flexible. The income of a takeaway rider depends on the number of orders finished. As to working hours, food delivery knights can also decide by themselves. According to the data of Meituan, 58.8% of takeaway riders work <4 h every day, but some riders choose to work more than 10 h. Full-time riders may choose to work more than 8 h in big cities. They tend to have a more flexible working time than construction workers. There are 36.2% of Meituan riders who spare <50% of their working hours on food delivery (Meituan, 2022a). Figure 7 shows the distribution of food delivery knights' working hour proportion.

The situation in the construction industry is completely different. For construction workers, although, in theory, they can work more for more pay and arrange their working hours by themselves, their workload is usually too heavy to achieve flexibility. Construction workers' daily working hours are always fixed by their foremen. Generally speaking, food delivery knights have more freedom during the work than workers in construction industries. The chance of more work for more pay and balancing work and life by themselves may be an important factor attracting young migrant workers to the food delivery business. Therefore, we hypothesize that young migrant workers have a stronger sense of autonomy.

### Hypothesis 3: The Working Environment Is More Important in Job Selection for Food Delivery Knights

Nowadays, most construction projects in China are located in suburban areas, and the accommodation for workers is poor. Besides, construction jobs usually require a long working time with high intensity, leading to a low appeal to job seekers (Jing-xiao et al., 2010). Food delivery knights can work and live in urban areas; the working hour and intensity are flexible. Studies also showed that the young generation in China cares more about life quality when choosing their job (Zhu et al., 2015). Therefore, we promote the hypothesis that the working environment may be a key factor that attracts young migrant workers.

### Hypothesis 4: Psychological Needs Are More Important in Job Selection for Food Delivery Knights

Psychological needs play an important role in job choice theory. Zhong et al. (2017) studied the impact of psychological contracts on job mobility in the construction industry, especially for migrant construction workers in China. Mucci et al. (2019) systematically stated the psychological health problem of migrant workers and mentioned that migrant workers in China suffered from great working pressure, and many had symptoms of psychological health problems. Koene and Paauwe mentioned that psychological contracts (PC) could strengthen and support each other in a specific workplace (Pot et al., 2001). Gellerman (1963) noted that the candidate would tend to choose an organization that they believe will most likely meet their psychological needs. Barlow (1965) reported that engineers' spiritual needs and expectations of their jobs would forge ahead.

As an emerging industry, the food delivery business has drawn society's attention and has been positively reported by the media in past years. During COVID-19, there is also news that considers some food delivery knights' work in a quarantined area as a “heroic” action. It is important to investigate whether attention and positive comments would satisfy the food delivery knights' psychological needs and attract more migrant workers. Herein, we hypothesized that food delivery knights show more concern for psychological needs in their job search behavior.

### Hypothesis 5: Personal Development Is More Important in Job Selection for Food Delivery Knights

Personal development has become an important job determinant for young migrant workers in recent years. Many chose their jobs not only for current needs but also for long-term development. On the one hand, migrant workers hardly get any promotion in the construction industry. Most of them have limited education levels. The long working time and heavy workload further prevent them from learning more skills for personal development. On the other hand, many food delivery knights only consider the job a springboard. Nevertheless, the food delivery business only emerged several years ago, and the career path is unclear. The flexible mechanism of working time offers the delivery knights an opportunity to learn more skills for other jobs. Therefore, we hypothesize that personal development is more important in food delivery knights' job selection.

### Hypothesis 6: Family Is More Important in Job Selection for Food Delivery Knights

Construction workers usually need to work and live on the construction site for a long time, and it is difficult for them to spare time with their families. Research also mentioned that the old generation of migrant workers in China did not pay more attention to their families (Liu et al., 2013; Gao et al., 2018; Siu and Unger, 2020). In contrast, food delivery knights can work close to their hometowns; many can even return home after work. Thus, it may be an important reason for attracting many young migrant workers. Thus, we hypothesized that family is more important in food delivery knights' job selection.

## Materials and Methods

The investigation was conducted using face-to-face interviews, web crawler data analysis, and World Value Survey data analysis.

### Face-to-Face Interview

We conducted a face-to-face questionnaire survey on migrant workers in Hangzhou, China. The target audiences are young migrant workers from the food delivery industry and construction industry. Each questionnaire contains questions about the interviewee's basic information, such as age, educational background, marital status, and questions about the determinants when they chose the job. The questionnaire considered six primary factors influencing a migrant worker's job selection determinants: income, job autonomy, working condition, psychological needs, personal development, and family. Several questions were set to concretize each factor. In total, the questionnaire has 25 questions. Interviewees can choose five levels of “Very important,” “Important,” “Neutral,” “Not important,” and “Not considered” to measure the significance of each factor in their determination to engage in this job. All three surveys were conducted offline in a face-to-face manner.

The survey on takeaway riders was conducted over several months, from April 2021 to July 2021. We interviewed hundreds of riders on the streets and got 203 valid questionnaires. All the interviewees are aged from 18 to 35, and the average age is 28.7. There are 197 male riders and 6 female riders. A total of 104 have a bachelor's degree, and 56 graduated from a high school. One hundred and thirteen of them were married, and 86 had at least one kid. Most interviewees work more than 6 h a day, and 54 have other part-time jobs. The food delivery job is the first job for only 43 riders in our survey, while 87 riders turn to the food delivery industry due to the eruption of COVID-19.

From July 2021 to September 2021, we surveyed migrant workers from the construction industry. The subjects are from several construction companies and construction pre-fabrication manufacturing plants. We came to their work unit and conducted a face-to-face questionnaire survey. We retrieved 154 questionnaires from construction companies and 112 questionnaires from construction pre-fab manufacturing plants.

Most of the food delivery industry interviewees are from Zhejiang and its neighboring provinces, while those in construction industries are mainly from central and western Provinces. Compared to food delivery knights, the quality of questionnaires from construction workers is relatively lower. Many of the questionnaires retrieved are valueless and invalid. A possible reason is the interviewees' educational level. A total of 133 of them have never gone to a high school. We found that many interviewees were illiterate or could not understand the questions during the survey. Some considered this a “test” and tried to fill in “correct answers” without listening to our explanation. Some kept asking other people or copying other people's answers. Therefore, we eliminated some questionnaires, detailed as follows:

- Questionnaires with more than 15% questions not answered. If more than 15% of answers cannot reflect the respondents' views, it can be judged as an invalid questionnaire (Ren et al., 2011).
- Questionnaires with the same answer for more than half of the questions. According to the empirical rule proposed by Curran, respondents who have the same answer for more than half-scale can be seen as careless or ill-intentioned (Curran, 2016).
- Questionnaires with illogical answers. There are cases like a respondent writing his age under the question of “educational background,” an interviewee who choose “Very important” for the determinants “stable salary” and “Flexible salary” at the same time.
- Questionnaires with highly similar answers to others. We consider that such questionnaires are not the respondent's answers and will not be included in the analysis.

Eventually, we collected 93 valid questionnaires for construction workers and 89 valid questionnaires for pre-fab manufacturing workers.

To analyze the data set and compare the two groups, we use Kolmogorov–Smirnov test to address the two groups of segments. Since the data of food delivery knights and construction industry workers are two independent samples, we choose the two-sample Kolmogorov–Smirnov test for the analysis. The Kolmogorov–Smirnov statistic quantifies a distance between the empirical distribution function of the sample and the cumulative distribution function of the reference distribution or between the empirical distribution functions of two samples. In a two-sample case, the null distribution of this statistic is calculated under the null hypothesis that the samples are drawn from the same distribution, and the distribution considered under the null hypothesis is continuous but is otherwise unrestricted. The two-sample K–S test is sensitive to differences in both location and shape of the empirical cumulative distribution functions of the two samples. To verify the difference between the two sets of number segments, we choose the significance level of 5%.

### Web Crawler Data Analysis

Since Wanderer emerged as the first recorded web crawler at the Massachusetts Institute of Technology in early 1993 (Menczer et al., 2004), crawler technology has undergone more than 20 years of development, and the technology has become increasingly diverse. In order to meet the diverse needs of different users, many types of crawler systems have been created and developed. Crawler technology is quickly used in search engines or other related websites to get the content and other data of data websites in time. The web crawler can automatically collect all accessible web pages and their contents by setting, and the collected data can be further processed by the search engine so that users can accurately obtain the required information the first time.

In this article, we crawled data from Chinese websites and social media, which can reflect young migrant workers' attitudes toward their job selection. These data include news and industry reports from several major domestic portal websites (e.g., Baidu, Sohu, Sina, and Netease) and comments of young migrant workers from Chinese social media (e.g., micro-blog, Know, and Tik Tok).

In the process of information collection, the search range URL is used as the target URL of the web crawler, and the requests library is used to download the target web page to obtain the instantiated object. The obtained information is parsed using the bs4 library to obtain the required information. The crawler collected more than 7,000 pieces of recruitment information. We preprocessed the selected samples to ensure the reliability of the data and the authenticity of the research.

Then we used the Term frequency-inverse document frequency (TF-IDF) to quantify the importance of some keywords and phrases in a document and assessed the priority of different job determinant factors. TF-IDF is a numerical statistic method intended to reflect how important a word is to a document in a collection or corpus (Rajaraman and Ullman, 2011). It is often used as a weighting factor in information retrieval, text mining, and user modeling searches. The TF–IDF value increases proportionally to the number of times a word appears in the document and is offset by the number of documents in the corpus that contain the word, which helps to adjust for the fact that some words appear more frequently in general.

### World Value Survey Data Analysis

We collected data from the World Values Survey in seven waves^1^ conducted in mainland China for analysis. The World Values Survey (WVS) is organized by the world values Institute, a non-profit organization established in Sweden and managed by the World Values Survey Association (WVSA). WVS is an international research project dedicated to scientific research on the social, political, economic, religious, and cultural values of the world's people. The overall goal is to analyze people's values, beliefs, and norms from a cross-border comparative and long-term perspective, covering various topics in sociology, politics, economics, anthropology, social psychology, public health, and other fields. The survey cycle is 5 years. WVS has conducted value surveys in more than 100 countries around the world.

World Values Survey designed different questionnaires according to each country's specific historical and cultural situation, covering different aspects of values measurement. It uses the questionnaire interview as the benchmark of empirical research to investigate the respondents' values topics such as economic development, gender equality, career incentive, democracy, social capital, political participation, environmental protection, and subjective wellbeing and their changes over time. The survey data and analysis results are open to social science researchers and the public.

Since WVS adopts a standardized survey scale in various countries and nationalities around the world, it not only provides a systematic and consistent standard data for the study of specific social problems but also supports people in considering the differences and historical changes in the values of different social groups in terms of economy, politics, and culture. Thus, people can make a cross-cultural horizontal comparison of different cultural values and a vertical comparison of cultural values of the same society over time. WVS provides a wealth of data to study the changing values of the Chinese people.

The WVS questionnaire included 258 variables. In this article, we excluded irrelevant variables and selected five independent social-demographic variables: income, autonomy, family, psychological needs, and working environment. The rates of interviewees who care about the five variables are collected to measure different groups' attitudes in job selection.

## Results

### Hypothesis 1: The Amount and Stability of Salary Are Less Important in Job Selection for Food Delivery Knights

We conducted a K-S test on the two data samples to analyze the questionnaire survey data. The result demonstrates a significant difference between the two groups in the index “Income.” In Table 2, “Most Extreme Difference” represents the largest difference between the two samples of data, “Absolute” represents the absolute value; “Positive” and “Negative” represent the positive and negative value; “Kolmogorov-Smirnov Z” is the test statistics; “Asymp. Sig. (2-tailed)” is the 2-tailed asymptotic significance. In this test, we selected a confidence interval of 95%. If the “Asymp. Sig. (2-tailed)” value is <0.05, the two data samples have significant differences.

**Table 2:** Kolmogorov–Smirnov (K-S) test results of the index “income”.

		**Income**
Most extreme difference	Absolute	0.578
	Positive	0.578
	Negative	0.000
Kolmogorov-Smirnov Z		1.231
Asymp. Sig. (2-tailed)		0.048

The web crawler data in Table 3 show the index weights determined based on the word frequency. The result implies that the index weights related to “Income” for construction workers are higher than those of food delivery knights. The WVS data shows that the proportion of construction workers who regarded “Income” as the important factor in job selection is 87%, while the proportion of food delivery knights is 73%. It also implies that construction workers valued income more than food delivery knights. In summary, Hypothesis 1 holds.

**Table 3:** Weights of index “income”.

**Primary**	**Group**	**Primary**	**Secondary**	**Weight**
**index**		**index weight**	**index**	
Income	Construction workers	0.536	High salary	0.322
			Stable salary	0.104
			Back pay	0.110
	Food delivery knights	0.404	High salary	0.232
			Stable salary	0.092
			Back pay	0.080

### Hypothesis 2: Job Autonomy Is More Important in Job Selection for Food Delivery Knights

The questionnaire survey analysis of the two groups demonstrates a significant difference in the index “Autonomy” (refer to Table 4). It indicates that the food delivery knights value job autonomy as a more important factor than the construction workers.

**Table 4:** Kolmogorov–Smirnov test results of the index “autonomy”.

		**Autonomy**
Most extreme difference	Absolute	0.681
	Positive	0.681
	Negative	0.000
Kolmogorov-Smirnov Z		1.459
Asymp. Sig. (2-tailed)		0.038

The web crawler data in Table 5 implies that the weights of indexes related to “Autonomy” for food delivery knights are higher than those of construction workers. Data from WVS indicates that the proportion of construction workers in China who value the factor “Autonomy” is 54%, and that of food delivery knights is 68%, which supports the finding above. In summary, Hypothesis 2 holds.

**Table 5:** Weights of index “autonomy”.

**Primary index**	**Group**	**Primary**	**Secondary**	**Weight**
		**index weight**	**index**	
Autonomy	Construction workers	0.143	Flexible hours	0.065
			Flexible salary	0.054
			Quick pay	0.034
	Food delivery knights	0.203	Flexible hours	0.079
			Flexible salary	0.069
			Quick pay	0.055

### Hypothesis 3: The Working Environment Is More Important in Job Selection for Food Delivery Knights

The questionnaire survey analysis of the two groups demonstrates a significant difference in the index “Working environment,” and the value of food delivery knights is higher than that of construction workers (refer to Table 6).

**Table 6:** Kolmogorov–Smirnov test results of the index “working environment”.

		**Working environment**
Most extreme difference	Absolute	0.707
	Positive	0.707
	Negative	0.000
Kolmogorov-Smirnov Z		1.573
Asymp. Sig. (2-tailed)		0.014

The web crawler data in Table 7 implies that the weights of indexes related to the “Working environment” for food delivery knights are higher than construction workers. The WVS data shows that the proportion of food delivery knights in China who regarded “Working environment” as the important factor in job selection is 74%, higher than that of construction workers (57%). In summary, Hypothesis 3 holds.

**Table 7:** Weights of index “working environment”.

**Primary**	**Group**	**Primary**	**Secondary**	**Weight**
**index**		**index weight**	**index**	
Working	Construction workers	0.213	Working hours	0.124
environment			Working intensity	0.057
			Easy job	0.032
	Food delivery knights	0.279	Working hours	0.183
			Working intensity	0.054
			Easy job	0.042

### Hypothesis 4: Psychological Needs Are More Important in Job Selection for Food Delivery Knights

The questionnaire survey analysis demonstrates no significant difference between the two groups in the index “Psychological needs” (refer to Table 8).

**Table 8:** Kolmogorov–Smirnov test results of the index “psychological needs”.

		**Psychological needs**
Most Extreme Difference	Absolute	0.217
	Positive	0.217
	Negative	0.000
Kolmogorov-Smirnov Z		0.634
Asymp. Sig. (2-tailed)		0.816

According to the web crawler data in Table 9, the weights of indexes “Social comments” and “Family comments” for food delivery knights are higher, but the weight of “Life value” for food delivery knights is lower. The WVS data presents similar values for the two groups, the proportion of construction workers in China who value the factor “Psychological needs” is 51%, and the proportion of food delivery knights is 53%. In summary, Hypothesis 4 does not hold.

**Table 9:** Weights of index “psychological needs”.

**Primary**	**Group**	**Primary**	**Secondary**	**Weight**
**index**		**index weight**	**index**	
Psychological	Construction workers	0.110	Social comments	0.032
needs			Family comments	0.036
			Life value	0.042
	Food delivery knights	0.136	Social comments	0.053
			Family comments	0.055
			Life value	0.028

### Hypothesis 5: Personal Development Is More Important in Job Selection for Food Delivery Knights

The questionnaire survey analysis shows no significant difference between the two groups in the index “Personal development” (refer to Table 10).

**Table 10:** Kolmogorov–Smirnov test results of the index “personal development”.

		**Personal development**
Most extreme difference	Absolute	0.327
	Positive	0.327
	Negative	0.000
Kolmogorov-Smirnov Z		0.812
Asymp. Sig. (2-tailed)		0.673

Web crawler data in Table 11 also indicates that the weights of indexes related to “Personal development” for food delivery knights are close to those of construction workers. The WVS data shows that the proportion of construction workers in China who regarded “Personal development” as the important factor in job selection is 66%, close to that of food delivery knights (61%). In summary, Hypothesis 5 does not hold.

**Table 11:** Weights of index “personal development”.

**Primary**	**Group**	**Primary**	**Secondary**	**Weight**
**index**		**index weight**	**index**	
Personal	Construction workers	0.082	Skill improvement	0.026
development
			Social relationship	0.022
			Promotion	0.034
	Food delivery knights	0.074	Skill improvement	0.021
			Social relationship	0.022
			Promotion	0.031

### Hypothesis 6: Family Is More Important in Job Selection for Food Delivery Knights

The questionnaire survey analysis of the two groups demonstrates a significant difference in the index “Family,” and the value of food delivery knights is higher than that of construction workers (refer to Table 12).

**Table 12:** Test results of the index “family”.

		**Family**
Most extreme difference	Absolute	0.732
	Positive	0.732
	Negative	0.000
Kolmogorov-Smirnov Z		1.415
Asymp. Sig. (2-tailed)		0.037

The web crawler data in Table 13 implies that the weights of indexes related to “Family” for food delivery knights are higher than construction workers. The WVS data shows that the proportion of food delivery knights who regarded “Family” as the important factor in job selection is 63%, higher than construction workers (47%). In summary, Hypothesis 6 holds.

**Table 13:** Weights of index “family”.

**Primary**	**Group**	**Primary**	**Secondary**	**Weight**
**index**		**index weight**	**index**	
Family	Construction workers	0.085	Close to home	0.036
			Take after family members	0.049
	Food delivery knights	0.134	Close to home	0.073
			Take after family members	0.061

## Data Interpretation and Discussion

The above data and analysis inform some new tendencies of employment psychology during young migrant workers' job selection. The analysis result of Hypothesis 1 implies that the construction workers attached more importance to income, and they tend to pursue a job with a high and stable salary. Hypotheses 2, 3, and 6 indicate that job autonomy, working environment, and family are the three critical factors influencing job selection of food delivery knights. The employment psychology of some young migrant workers has experienced the change that income is no longer the single dominant factor in job selection. These new tendencies can explain the construction industry's labor shortage and aging problem. In the past decades, the living standards of urban and rural residents in China have improved. The new generation of migrant workers who grow up in this relatively affluent environment tends not to desperately pursue a high and stable salary like the older generation. With a weaker desire for income, young migrant workers can pay more attention to other factors like job autonomy, working environment, and family.

Despite the payment, workers suffer from high working intensity, harsh environment, lack of freedom, and very few opportunities for family life in the construction industry. These factors may keep many young migrant workers away. In contrast, a food delivery job can attract young men because of its flexible working mechanism, better working environment, and convenience for family life. Naturally, the food delivery industry is not the only one. Many other emerging businesses have similar characteristics and attract many young migrant workers. It also rings an alert for many traditional industries. The labor shortage and aging problems may continue for a long time, and it is no longer a problem that can be “easily” solved by raising salaries.

Both Hypotheses 4 and 5 do not hold. No significant difference can be found in the two group's employment psychology regarding psychological needs and personal development. It is well-known that neither a construction nor food delivery company provides the young migrant workers a formal career path, and they both have a low threshold for education and skill requirements. Speculation is that such characteristics attract young migrant workers who do not possess a strong need for psychological satisfaction or personal development.

Another question raised based on the speculation is whether such a phenomenon exists in the entire young generation of migrant workers or only in those in the investigated businesses. It was found that the interviewees in the two groups have different personal backgrounds. Especially the food delivery knights have higher education levels on average. Many migrant workers who work in the construction industry may have a hard time working for food delivery because they do not know how to use a phone app, while the food delivery knights may have a good chance to find another type of job. If even the food delivery knights demonstrated a low need for psychological satisfaction or personal development, we have a reason to assume this phenomenon exists in the young generation of migrant workers. Nevertheless, it requires further investigation to validate the hypothesis.

## Conclusion

In this article, we studied the employment psychology of young migrant workers in China. With data from a questionnaire survey, web crawler, and World Value Survey during the period of COVID-19, a comparative analysis was conducted between construction workers and food delivery knights. Results show that food delivery knights valued more about job autonomy, working environment, and family in job selection while construction workers valued more about income. The two groups showed similar attitudes to psychological needs and personal development. A new tendency of employment psychology has revealed that many young migrant workers do not desperately pursue good salaries but pay more attention to freedom, life, and family when selecting a job. The new tendency of young migrant workers' employment psychology can partly explain the labor shortage and aging in construction industries. The limitation of the study is that the investigated area is only restricted to Hangzhou city. Moreover, some data from web crawl may not precisely reflect young migrant workers' attitudes.

## Data Availability Statement

The original contributions presented in the study are included in the article/supplementary material, further inquiries can be directed to the corresponding author.

## Ethics Statement

Ethical review and approval was not required for the study on human participants in accordance with the local legislation and institutional requirements. Written informed consent for participation was not required for this study in accordance with the national legislation and the institutional requirements.

## Author Contributions

CX: writing the draft, methodology, and analysis. CZ: draft editing. XS: conceptualization, editing, and supervision. ZQ: data analysis and draft review. CZ and CX: data acquisition. All authors contributed to the article and approved the submitted version.

## Conflict of Interest

The authors declare that the research was conducted in the absence of any commercial or financial relationships that could be construed as a potential conflict of interest.

## Publisher's Note

All claims expressed in this article are solely those of the authors and do not necessarily represent those of their affiliated organizations, or those of the publisher, the editors and the reviewers. Any product that may be evaluated in this article, or claim that may be made by its manufacturer, is not guaranteed or endorsed by the publisher.

## Footnotes

^1^https://www.worldvaluessurvey.org/WVSContents.jsp.
